# District-Level Risk Mapping of Highly Pathogenic Avian Influenza in Poultry in Kazakhstan Using a Multi-Criteria Decision Model

**DOI:** 10.3390/pathogens15080796

**Published:** 2026-07-27

**Authors:** Aizada A. Mukhanbetkaliyeva, Irene Iglesias Martin, Fedor I. Korennoy, Alimzhan S. Kadyrov, Yersyn Y. Mukhanbetkaliyev, Saidulla I. Ruzmatov, Asem Zh. Abenova, Temirlan G. Bakishev, Andres M. Perez, Sarsenbay K. Abdrakhmanov

**Affiliations:** 1 Institute of Animal Science and Veterinary, Saken Seifullin Kazakh Agrotechnical University, Astana 010011, Kazakhstan; aizada.1970@mail.ru (A.A.M.); kadyrov.alike@gmail.com (A.S.K.); ersyn_1974@mail.ru (Y.Y.M.); saidulla.ruzmatov@mail.ru (S.I.R.); asem.abenova.1993@mail.ru (A.Z.A.); bakishevt@mail.ru (T.G.B.); 2 Centro de Investigación en Sanidad Animal (CISA), Instituto Nacional de Investigación y Tecnología Agraria y Alimentaria (INIA), Consejo Superior de Investigaciones Científicas (CSIC), 28130 Madrid, Spain; iglesias@inia.csic.es; 3 Federal Centre for Animal Health (FGBI ARRIAH), Vladimir 600901, Russia; korennoy@arriah.ru; 4 Center for Animal Health and Food Safety, College of Veterinary Medicine, University of Minnesota, St. Paul Campus, St. Paul, MN 55108, USA; aperez@umn.edu

**Keywords:** highly pathogenic avian influenza (HPAI), risk mapping, multi-criteria decision analysis (MCDA), TOPSIS, poultry, wild birds, Kazakhstan, spatial epidemiology, surveillance, migratory flyways

## Abstract

Highly pathogenic avian influenza (HPAI) is a transboundary disease of birds with zoonotic potential. Since 2021, H5 HPAI virus variants have spread globally, with migratory waterfowl playing a key role in transcontinental dissemination. Kazakhstan lies at the intersection of major flyways, creating a persistent risk of virus introduction into domestic poultry. While HPAI risk in wild birds has been explored in Kazakhstan, the spatial risk for poultry has remained underassessed. Here, we present a risk map for HPAI in poultry across 174 administrative districts of Kazakhstan, using a multi-criteria decision analysis (TOPSIS) that integrates five quantitative risk indicators that relate to wild bird habitat, virus survival in the environment and poultry farm census. The obtained district-level risk index agreed well with historical outbreak data (2005–2025), with an area under the receiver operating characteristic (ROC) curve of 0.899 (95% CI: 0.846–0.952). Most districts (56%) were at negligible risk, whereas medium- and high-risk clusters occurred in the north, central, and southern regions, and near the Caspian Sea. These findings provide an operational, spatially explicit tool for targeting surveillance and biosecurity measures to high-risk areas, ultimately helping to mitigate the impact of one of the most devastating transboundary poultry diseases in Central Asia.

## 1. Introduction

Highly pathogenic avian influenza (HPAI) is a highly contagious, transboundary disease of domestic, synanthropic, and wild birds, occurring in the form of epizootics or enzootics, characterized by damage to the respiratory organs and gastrointestinal tract, general depression, and decreased productivity. The causative agents of the disease are viruses of the species *Alphainfluenzavirus influenzae* (commonly known as influenza A viruses), family *Orthomyxoviridae*, among which the H5 and H7 subtypes have the greatest epidemiological significance [1,2,3,4]. Highly pathogenic variants are characterized by high infectiousness and mortality among poultry, reaching 90–100% under certain conditions, which leads to significant economic losses and the need for mass culling of susceptible domestic populations [5]. In addition, some virus variants are capable of infecting mammals, including humans [6,7,8].

Since 2021, a panzootic HPAI infection has been observed globally, caused mainly by variants of the H5 virus. In contrast to earlier epizootics, modern virus strains are increasingly circulating in migratory bird populations, thereby contributing to the transcontinental spread of infection [9,10,11,12]. In particular, from 2021 to 2024, HPAI outbreaks were registered on almost all continents. For example, in 2023, 157 HPAI outbreaks were reported in Europe, Asia, and the Americas, 37 of which were in poultry [13]. In total, about 2.5 million poultry were destroyed, and in all cases the H5N1 subtype prevailed [14,15]. Furthermore, HPAI was registered in 108 countries on five continents in 2024, causing the deaths of more than 300 million birds worldwide, as the virus continues to overpass inter-species barriers [16]. In 2025, the high epizootic activity of HPAI also persisted, as evidenced by more than 7 thousand outbreaks among domestic and wild birds in 75 countries worldwide, accompanied by the death or forced destruction of poultry in excess of 98 million [17].

In such a context, Kazakhstan remains vulnerable to HPAI epidemics. Kazakhstan, the largest landlocked country in the world, is geographically located at the very center of the Eurasian continent, which determines an important role in the migration of wild migratory birds. Three global bird flightways run through the country. The Central Asian Flyway (CAF) connects Siberian and Arctic breeding grounds with wintering grounds in India, Pakistan, the Persian Gulf, and East Africa. The East African–West Asian Flyway covers the western regions of the country, intersecting with the CAF near the northern coast of the Caspian Sea. The East Asian-Australasian flyway (East Asian–Australasian Flyway) affects only the eastern regions of the country [18,19]. In addition to these main migration flyways of migratory birds, an equally important local “Siberian-Caspian” flyway passes through the territory of Kazakhstan, which connects breeding grounds in Western Siberia with wintering and molting sites on the northern and eastern coasts of the Caspian Sea. This flyway is of particular importance in bringing HPAI into the Caspian region [20].

Kazakhstan is, arguably, one of the most important reserves for waterfowl in Asia, with 130 bird species registered in the republic during nesting, molting, seasonal migrations, and wintering. Every year, the number of nesting bird species reaches 10 million, 2–3 million birds molt, and about 50 million migratory birds stop in the country’s reservoirs during spring and autumn migrations, which is why the Tengiz–Korgalzhyn lake systems are included in the Ramsar List of Wetlands of International Importance. Wetland birds are numerous and diverse here and are represented by 112 species, which make up 87% of the avifauna of the wetlands of Kazakhstan [21]. During the migration season, the population density on key lakes (Tengiz, Korgalzhyn, Koibagar, Alakol, Balkhash, Markakol, and North Caspian) increases tenfold, which creates ideal conditions for the contact exchange of viruses between species [21]. With such a large density of waterfowl in proximity to industrial poultry facilities, the risk for introduction of the virus into commercial farms is not negligible. Certainly, it has been demonstrated that outbreaks of HPAI in poultry are spatially related to the proximity of water bodies or the presence of wild birds [22,23,24]. In the Netherlands, it was noted that during outbreaks of HPAI in the autumn and winter of 2016–2017, the density of wild waterfowl on poultry located near wetlands was significantly higher compared to uninfected poultry concentrated in areas with insufficient water [24].

For Kazakhstan, the problem of HPAI has remained relevant over the past two decades. Since 2005, outbreaks of this disease have occurred in the country approximately every 3–4 years, leading to significant mortality among poultry populations [25,26]. At the same time, the situation escalated dramatically in 2020, when an incursion of the H5N8 HPAI virus clade 2.3.4.4b rapidly spread throughout the country, affecting 9 of the 14 regions of Kazakhstan. In total, 1.9 million birds died during that period, representing 4.2% of the total poultry population of the country (45 million birds) [27]. These epizootics have shown a high vulnerability of individual territories of the country to the introduction and spread of the virus due to contacts between wild and domestic poultry [28].

Despite the available data on the spread and circulation of HPAI viruses in Kazakhstan, most of the available studies are aimed at studying the virological and molecular genetic characteristics of the pathogen and the epidemiological features of the disease [20,25,26,28]. Additionally, the role of certain species of migratory birds in maintaining the circulation of the virus and determining the priorities of epidemiological surveillance has also been assessed [20,29,30,31]. However, the spatial distribution of the risk for occurrence and spread of HPAI outbreaks in poultry farms remains unclear.

The present study aims at addressing this gap by applying a multi-criteria decision analysis (TOPSIS, Technique for Order Preference by Similarity to Ideal Solution) to estimate the relative risk for each of the 174 administrative districts of Kazakhstan, explicitly targeting the domestic bird population. Results will help design surveillance and biosecurity programs specifically targeting areas estimated to be at highest risk for the disease. Ultimately, these results will help inform decisions intended to prevent or mitigate the impact of one of the most impactful transboundary animal diseases of poultry in Central Asia.

## 2. Materials and Methods

### 2.1. General Approach

The Republic of Kazakhstan holds the ninth place in the world in terms of geographic area. Administratively, the country is divided into 17 first-level units (oblasts) and 174 second-level units (districts). The domestic poultry sector is heterogeneous across the country. According to recent data, Almaty, East Kazakhstan, and Akmola oblasts together produce over 75% of Kazakhstan’s poultry meat, indicating a non-random spatial distribution of poultry production that is likely to influence disease dynamics [32]. The study was designed to develop a spatially explicit district-level risk map for HPAI occurrence in domestic poultry. This spatial resolution was selected to align with data availability and to provide an operational scale for veterinary decision-making and resource allocation. The risk assessment was conceptualized as a Multi-Criteria Decision Analysis (MCDA) problem, integrating variables related to environmental hazard, wildlife exposure, and poultry sector vulnerability [33]. All calculations were performed using Microsoft Excel, included in Microsoft Office LTSC Professional Plus 2024 (Microsoft Corporation, Redmond, WA, USA), and results were depicted using ArcGIS Pro 3.5.3 (Esri, Redlands, CA, USA) to produce the final HPAI risk maps.

### 2.2. Selection and Processing of Risk Indicators

Five quantitative risk variables were selected to characterize the epidemiological landscape of each district. These variables represented extrinsic (environmental and wildlife-related) and intrinsic (related to poultry farm demographics) factors expected to influence the risk for the disease.

i. Priority wild bird observation density: Migratory waterfowl act as the primary natural reservoir for HPAI viruses. To quantify this risk, citizen science data from the eBird Basic Dataset (EBD) for the period 2019–2023 were utilized [34]. Observations of 137 priority risk species, previously identified based on their susceptibility and involvement in HPAI outbreaks in Kazakhstan [30], were extracted. A kernel density estimation (KDE) was performed to evaluate the spatial concentration of these observations. KDE was implemented using the standard Kernel Density tool in ArcGIS Pro, which applies a quartic kernel, with a fixed search radius (bandwidth) of 3° and the planar distance method. The mean density value was subsequently calculated for each district using zonal statistics tool in ArcGIS Pro.
ii. Wild bird census in wetlands: Wetlands serve as critical ecological interfaces for pathogen transmission between wild birds and domestic poultry [24,35]. A national wetlands database [36,37] was used to identify water bodies. The annual mean number of observations for risk species recorded in the eBird database within a 10-m buffer of these wetlands was calculated and aggregated at the district level to represent the local magnitude of the wildlife–environment interface.
iii. Environmental virus survival (days): Virus persistence in the environment decreases as temperature increases, with low temperatures extending the window for indirect transmission through contaminated water or surfaces [38]. Meteorological raster data providing monthly minimum temperatures were extracted from ERA5 Google and Copernicus Data Store and processed within Kazakhstan boundary [39]. The mean minimum temperature for each district was extracted using zonal statistics in ArcGIS Pro software. To transform this temperature into an estimate of virus survival time, a logarithmic regression function previously validated in early warning tools was applied [40]:
  t(T) = −7.82 ln(T) + 29.94, where t is the virus survival time in days, and T is the temperature in degrees Celsius. A truncation rule was applied for temperatures below 1 °C to avoid numerical inconsistencies while reflecting maximum persistence under freezing conditions.
iv and v. Poultry farm counts and farm capacity: The structure and density of the poultry production system are critical determinants of HPAI spread and amplification. Higher densities increase transmission risk due to greater contact rates and connectivity, while farm capacity influences outbreak probability and magnitude [41,42]. Georeferenced data on the location and capacity of poultry farms in Kazakhstan were provided by the national veterinary authorities [43]. These data were spatially joined to the district boundaries to calculate two distinct variables per district, namely, the total number of poultry farms (farm counts) and the total number of domestic birds (farm capacity).

### 2.3. Multi-Criteria Decision Analysis Using TOPSIS

An analytical method referred to as Technique for Order of Preference by Similarity to Ideal Solution (TOPSIS) was used to compute a single risk index for each district based on the values of the five risk variables described above [44]. TOPSIS is an MCDA method that ranks alternative solutions based on the concept that the optimal solution should have the shortest geometric distance to the positive ideal solution (Ri+) and the longest geometric distance to the negative ideal solution (Ri−). In the context of HPAI risk assessment, the Ri+ represents a hypothetical district with the highest possible risk across all variables, whereas the Ri-represents a district with the lowest possible risk [45].

The TOPSIS methodology was executed through the following sequential steps:

(a) a normalized decision matrix (dimensions districts × criteria/variable) was specified.
(b) Each criterion column was multiplied by a weight factor that represents the relative contribution of the variable to the risk index and reflects its epidemiological importance, thereby producing a weighted normalized matrix.
(c) Ri+ and Ri− were identified based on the maximum and minimum estimated alternative, respectively.
(d) For each district (i), the Euclidean distances to Ri+ (Di^+) and Ri− (Di^−) were computed across all criteria.
(e) The TOPSIS score for each district i (Ci) was computed as Di^−/(Di^+ + Di^−), so that Ci represents a composited estimate of risk for the district with extreme values of 0 (no risk) and 1 (highest possible risk).

### 2.4. Weighting Schemes

The weights used by Spain to predict the risk for HPAI outbreaks were also used here [40]. In that early work, briefly, a structured expert-driven approach referred to as Analytic Hierarchy Process (AHP) was used to derive weights through pairwise comparisons [46]. The relative importance of each pair of indicators was evaluated on a scale from 1 (equal importance) to 9 (extreme importance) based on the available scientific evidence regarding the role of each factor in HPAI introduction and spread, as described in the literature cited for each risk indicator in the previous section. The relative importance of each pair of indicators was evaluated on a scale from 1 (equal importance) to 9 (extreme importance). Priority wild bird observation density was assigned the highest priority, being considered 5/4 times more important than farm count and wild bird census in wetlands, 8/5 times more important than poultry census, and 5/2 times more important than virus survival. Farm count and wild bird census in wetlands were judged equally important (ratio = 1), and both were considered 4/3 times more important than poultry census and twice as important as virus survival. The resulting pairwise comparison matrix yielded the following normalized weights: Priority wild bird observation density (0.276), Farm count (0.222), Wild bird census in wetlands (0.222), Poultry census (0.168), and Environmental virus survival (0.111) (Table 1).

### 2.5. Model Validation and Mapping

Model results were validated using historical outbreak data. Districts that reported HPAI outbreaks to the World Organisation for Animal Health (WOAH) during a 20-year period (2005–2025) were considered cases, and as controls otherwise. The receiver operating characteristic (ROC) curve was computed using the risk index predicted for each district. ROC-curve analysis was performed in the software environment for statistical computing and graphics R using the pROC package [47]. To determine the optimal risk threshold that maximizes the overall predictive performance, the Youden index (J = sensitivity + specificity − 1) was computed from the ROC curve [48]. The cut-off value corresponding to the maximum Youden index was selected as the optimal decision boundary, balancing the trade-off between true positive and false positive rates.

For cartographic visualization only, the continuous risk predicted score was categorized into four categories, referred to as negligible, low, medium, and high, using Jenks natural breaks optimization method [49,50]. This data-driven method minimizes within-class variance and maximizes between-class differences, making it well-suited for skewed risk distributions typical in epidemiological data. The categorized scores were not used in the TOPSIS calculation or ROC analysis.

## 3. Results

**Figure 1:**
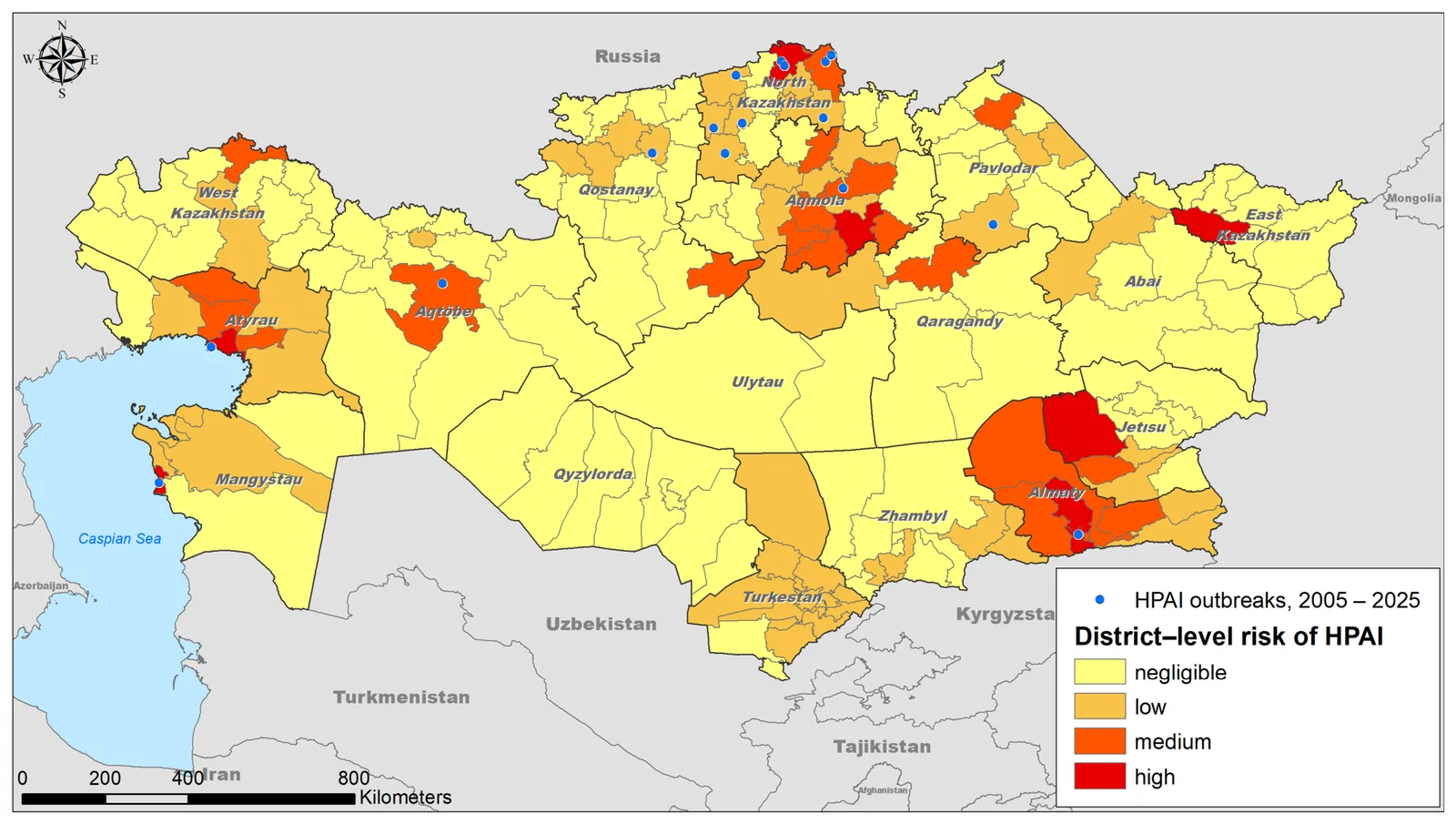
District-level risk of highly pathogenic avian influenza (HPAI) estimated in Kazakhstan. Risk was categorized as negligible (yellow), low (light orange), medium (dark orange), and high (red). Blue dots indicate HPAI outbreaks between 2005 and 2025.

Predicted risk was negligible (risk score < 0.058) for most (n = 98, 56%) districts throughout Kazakhstan. Medium-risk districts (n = 21, 12%) and high-risk districts (n = 8, 5%) were mainly clustered in the northern, central, and southern regions of Kazakhstan, as well as near the Caspian Sea. The risk score ranges for these groups were 0.166–0.315 and 0.316–0.520, respectively (Figure 1).

The predicted risk aligned well with the districts that reported outbreaks between 2005 and 2025, as indicated by the area under the curve (AUC = 0.899, 95% CI: 0.846–0.952) estimated for the resulting ROC. Using the Youden index, the optimal risk threshold was identified as 0.12, corresponding to a sensitivity of 1.0 and specificity of 0.80.

## 4. Discussion

The presented study summarizes the district-level risk predicted for poultry farms in the Republic of Kazakhstan using a combination of factors reported in the peer-reviewed literature to influence that risk, weighted by their relative importance. Our findings are consistent with risk mapping studies conducted in other regions where migratory flyways intersect with intensive poultry production. For instance, in The Netherlands, Velkers et al. [24] demonstrated that the density of wild waterfowl near poultry farms was a significant predictor of HPAI outbreaks during autumn migration—a pattern we also observed in the northern and central districts of Kazakhstan. Similarly, Gilbert and Pfeiffer [22], in a global review, identified proximity to wetlands and poultry density as recurrent risk factors across Southeast Asia and Europe, supporting the structure of our MCDA model. However, unlike the fragmented agricultural landscapes of Western Europe, where farm densities are uniformly high, Kazakhstan exhibits a more polarized pattern: extensive low-risk areas interspersed with high-risk clusters, likely due to the concentrated nature of its poultry industry. Comparable TOPSIS-based approaches have been applied in Canada for avian influenza surveillance [45] and in Spain for HPAI early warning [40]. While those studies also prioritized wild bird-related variables, the way temperature-dependent virus survival is incorporated differs: in the Spanish DiFLUsion system, survival time directly multiplies the risk score, whereas in our AHP-based model it is treated as one of five weighted criteria and received the lowest weight (0.111). This difference likely reflects Kazakhstan’s more continental climate, where prolonged freezing temperatures may limit environmental transmission during winter, reducing the relative importance of this factor compared to regions with milder winters.

Most (56%) districts were estimated at negligible risk, which is likely a consequence of the heterogeneous distribution of poultry production in Kazakhstan, with a large concentration of operations in some specific regions. For that reason, even if the country occupies a strategic geographical position at the intersection of the Central Asian, West Asian–East African, and Black Sea–Mediterranean migratory flyways [29] and its extensive wetlands and steppes support populations of tens of millions of migratory waterbirds, making it a critical node for the intercontinental transmission of HPAI viruses [30], the domestic-wild bird interface is relatively clustered in the country.

**Figure 2:**
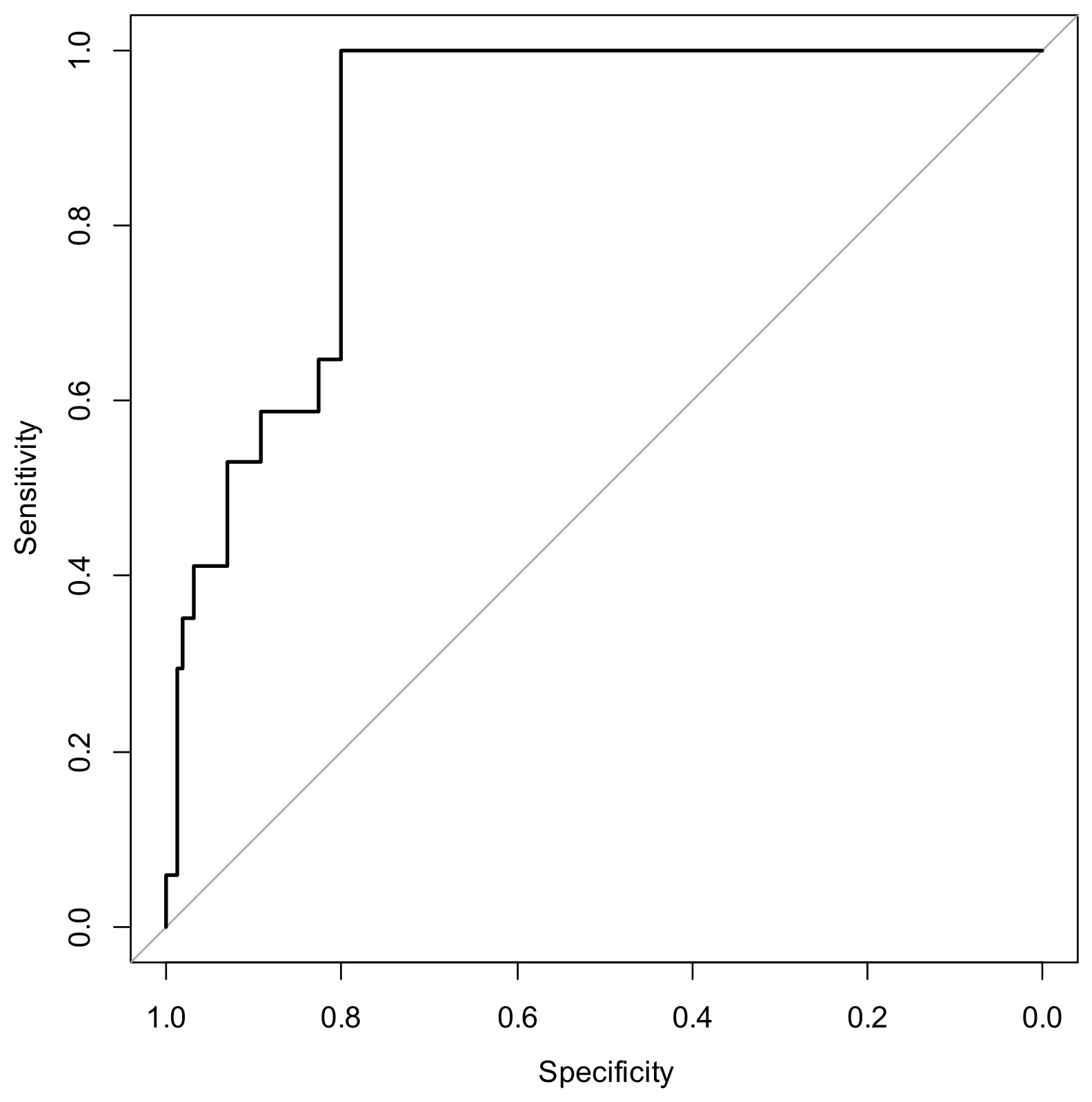
Receiver Operating Characteristic (ROC) curve of the predicted district-level risk of highly pathogenic avian influenza (HPAI) in Kazakhstan.

The use of a cut-off value of 0.12 on the predicted scores resulted in values of Se = 1 and Sp = 0.8 (Figure 2), indicating that such a threshold would be able to accurately predict the risk for all the districts that reported HPAI outbreaks between 2005 and 2025. This high sensitivity, also observed in other MCDA-based risk models [40,45], supports the utility of the TOPSIS framework for operational early warning, though it also suggests that the model may be over-sensitive to the input variables, potentially generating false positives in districts with no historical outbreaks. Future prospective validation using upcoming outbreak data would help refine the optimal threshold.

A key strength of our approach is its operational scale. By producing risk estimates at the district level—the same administrative unit used by veterinary services for resource allocation—the map can be directly integrated into existing surveillance and biosecurity programmes. For example, districts in the high-risk stratum (e.g., in Akmola, North Kazakhstan, and West Kazakhstan oblasts) should prioritize active surveillance, wild bird monitoring near wetlands, and biosecurity audits of poultry farms before and during migration seasons. Conversely, districts with negligible risk may maintain routine passive surveillance, allowing efficient use of limited veterinary resources.

**Figure 3:**
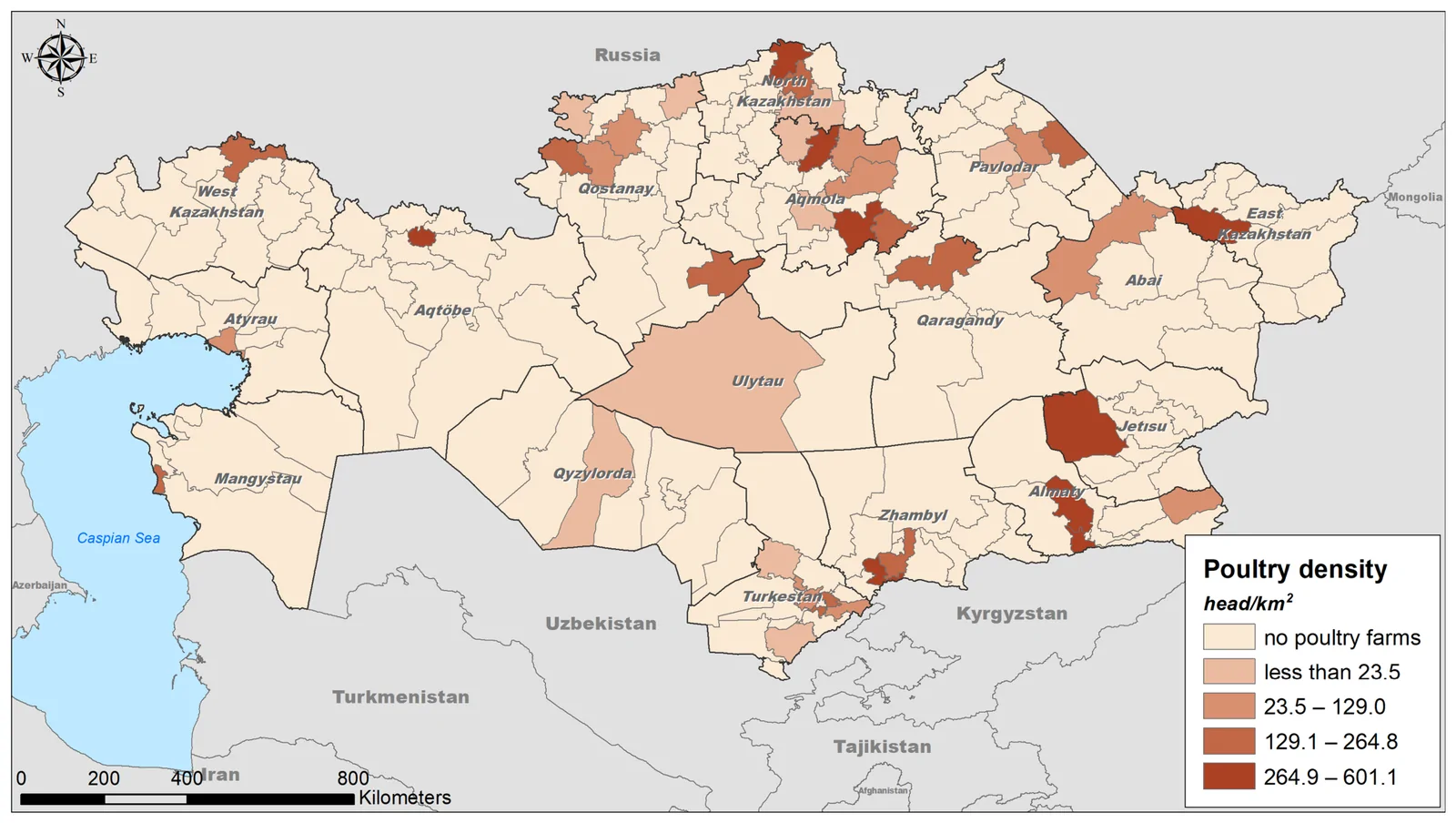
District-level distribution of poultry density in the Republic of Kazakhstan.

To enable readers to assess whether high-risk districts coincide with areas of high poultry population density, we provide a map (Figure 3) showing the spatial distribution of poultry population density across Kazakhstan. High-risk districts in the north and south-east generally coincide with areas of high poultry density. Near the Caspian Sea, however, high-risk districts show only moderate poultry densities, and many medium-risk districts in central Kazakhstan have few or no poultry farms. This pattern suggests that wild birds and their habitats are the main drivers of risk in some regions, while in others the risk is amplified by poultry concentration. Consequently, control strategies should differ: farm-level biosecurity and audits are more relevant in densely populated poultry areas, whereas wild bird monitoring and wetland management should be prioritised in low-density high-risk regions.

One important limitation of this work is the assumption that the weights that were used for Spain also apply to Kazakhstan. Future studies should aim to elicit weights from local experts using the same AHP framework to account for country-specific epidemiological and production realities. A sensitivity analysis of the TOPSIS rankings to variations in the weight vector would also be a valuable extension. Additionally, while our model integrates five key variables, other potentially relevant factors—such as farm biosecurity levels, trade networks, or the presence of live bird markets—were not included due to data unavailability. Incorporating these in future iterations could enhance the model’s specificity.

In conclusion, the work here presents, for the first time, a district-level map of the predicted risk for HPAI occurrence in poultry in Kazakhstan based on the evaluation of risk factors. The results will be fundamental in informing the allocation of resources to support surveillance and control activities in the country. Ultimately, the results will contribute to the prevention and control of one of the most important transboundary diseases of poultry in Central Asia. The methodology presented here could also be helpful to inform the characterization of risk in other countries in the region.

## Figures and Tables

**Table 1:** Pairwise comparison of the relative importance of factors influencing the risk for highly pathogenic avian influenza (HPAI) in Kazakhstan, along with the resulting Analytic Hierarchy Process (AHP) weight, as adopted from evaluations in Spain, that was used in a predictive model for the country.

	Priority Wild Bird Observation Density	Farm Count	Wild Bird Census (Wetlands)	Poultry Census	Virus Survival (Days)	AHP Weight
Priority wild bird observation density	1	5/4	5/4	8/5	5/2	0.276
Farm count	4/5	1	1	4/3	2	0.222
Wild bird census (wetlands)	4/5	1	1	4/3	2	0.222
Poultry census	5/8	3/4	3/4	1	3/2	0.168
Virus survival (days)	2/5	1/2	1/2	2/3	1	0.111

## Data Availability

The data used in the present research (specifically, HPAI outbreaks location data and environmental layers) are publicly available, and their sources are listed.

## References

[1] Alexander D.J. (2009). Avian Influenza and Newcastle Disease: A Field and Laboratory Guide.

[2] Onischenko G.G., Netesov S.V., Agafonov A.P., Safatov A.S., Buryak G.A., Generalov V.M., Sergeyev A.N., Drozdov I.G. (2006). Highly pathogenic avian influenza: A new pandemic threat and possibilities to resist it. Vestn. Ross. Akad. Meditsinskikh Nauk..

[3] De Bruin A.C.M., Funk M., Spronken M.I., Gultyaev A.P., Fouchier R.A.M., Richard M. (2022). Hemagglutinin Subtype Specificity and Mechanisms of Highly Pathogenic Avian Influenza Virus Genesis. Viruses.

[4] Shi J., Zeng X., Cui P., Yan C., Chen H. (2023). Alarming situation of emerging H5 and H7 avian influenza and effective control strategies. Emerg. Microbes Infect..

[5] Guo F., Li Y., Yu S., Liu L., Luo T., Pu Z., Xiang D., Shen X., Irwin D.M., Liao M. (2019). Adaptive evolution of human-isolated H5Nx avian influenza a virus. Front. Microbiol..

[6] Burrough E.R., Magstadt D.R., Petersen B., Timmermans S.J., Gauger P.C., Zhang J., Siepker C., Mainenti M., Li G., Thompson A.C. (2024). Highly Pathogenic Avian Influenza A(H5N1) Clade 2.3.4.4b Virus Infection in Domestic Dairy Cattle and Cats, United States, 2024. Emerg. Infect. Dis..

[7] Webby R.J., Uyeki T.M. (2024). An Update on Highly Pathogenic Avian Influenza A(H5N1) Virus, Clade 2.3.4.4b. J. Infect. Dis..

[8] Krammer F., Hermann E., Rasmussen A.L. (2025). Highly pathogenic avian influenza H5N1: History, current situation, and outlook. J. Virol..

[9] The World Organization for Animal Health (WOAH) Avian Influenza. https://www.woah.org/en/disease/avian-influenza/.

[10] World Health Organization Assessment of Risk Associated with Recent Influenza A(H5N1) Clade 2.3.4.4b Viruses. https://www.who.int/publications/m/item/assessment-of-risk-associated-with-recent-influenza-a(h5n1)-clade-2.3.4.4b-viruses.

[11] Islam A., Hossain M.E., Amin E., Islam S., Islam M., Sayeed M.A., Hasan M.M., Miah M., Hassan M.M., Rahman M.Z. (2023). Epidemiology and Phylodynamics of Multiple Clades of H5N1 Circulating in Domestic Duck Farms in Different Production Systems in Bangladesh. Front. Public Health.

[12] Updated Joint FAO/WHO/WOAH Public Health Assessment of Recent High Pathogenicity Avian Influenza A(H5) Virus Events in Animals and People. https://www.woah.org/app/uploads/2026/05/2026-05-18-fao-woah-who-h5-assessment.pdf.

[13] Organización Mundial de Sanidad Animal (2023). Distribution of HPAI New Outbreaks in Poultry, and Corresponding Subtypes.

[14] Organización Mundial de Sanidad Animal (2023). Influenza Aviar—WOAH—Organización Mundial de Sanidad Animal.

[15] Agüero M., Monne I., Sanchez A., Zecchin B., Fusaro A., Ruano M.J., del Valle Arrojo M., Fernandez-Antonio R., Souto A.M., Tordable P. (2023). Highly pathogenic avian influenza A(H5N1) virus infection in farmed minks, Spain, October 2022. Eurosurveillance.

[16] United Nations Avian Flu Reported in 108 Countries Across Five Continents, Says UN Health Agency. https://news.un.org/en/story/2024/12/1158286.

[17] Enikeeva A.I., Panova A.S., Vasiltsova N.N., Danilenko A.V., Shadrinova K.N., Svyatchenko S.V., Ivanova K.I., Onkhonova G.S., Goncharova N.I., Tregubchak T.V. (2026). Review of the Epizootic and Epidemic Situation regarding Highly Pathogenic Avian Influenza in Russia and Globally in 2025. Probl. Part. Danger. Infect..

[18] Nagy S., Langendoen T. (2020). Flyway Trend Analyses Based on Data from the African-Eurasian Waterbird Census from the Period of 1967–2018.

[19] Si Y., Skidmore A.K., Wang T., De Boer W.F., Debba P., Toxopeus A.G., Li L., Prins H.H.T. (2009). Spatio-temporal dynamics of global H5N1 outbreaks match bird migration patterns. Geospat. Health.

[20] Kydyrmanov A., Sayatov M., Karamendin K., Zhumatov K., Asanova S., Daulbayeva K., Starick E., Fereidouni S. (2017). Monitoring of Influenza A Viruses in Wild Bird Populations in Kazakhstan in 2002–2009. Arch. Virol..

[21] Schielzeth H., Eichhorn G., Heinicke T., Kamp J., Koshkin M.A., Koshkin A.V., Lachmann L. (2008). Waterbird population estimates for a key staging site in Kazakhstan: A contribution to wetland conservation on the Central Asian flyway. Bird Conserv. Int..

[22] Gilbert M., Pfeiffer D.U. (2012). Risk factor modelling of the spatio-temporal patterns of highly pathogenic avian influenza (HPAIV) H5N1: A review. Spat. Spatiotemporal Epidemiol..

[23] Si Y., de Boer W.F., Gong P. (2013). Different Environmental Drivers of Highly Pathogenic Avian Influenza H5N1 Outbreaks in Poultry and Wild Birds. PLoS ONE.

[24] Velkers F.C., Manders T.T.M., Vernooij J.C.M., Stahl J., Slaterus R., Stegeman J.A. (2021). Association of Wild Bird Densities around Poultry Farms with the Risk of Highly Pathogenic Avian Influenza Virus Subtype H5N8 Outbreaks in the Netherlands, 2016. Transbound. Emerg. Dis..

[25] Sultankulova K., Argimbayeva T., Aubakir N., Bopi A., Omarova Z., Melisbek A., Karamendin K., Kydyrmanov A., Chervyakova O., Kerimbayev A. (2024). Reassortants of the Highly Pathogenic Influenza Virus A/H5N1 Causing Mass Swan Mortality in Kazakhstan from 2023 to 2024. Animals.

[26] Kydyrmanov A., Karamendin K., Kassymbekov Y., Daulbayeva K., Sabyrzhan T., Khan Y., Nuralibekov S., Baikara B., Fereidouni S. (2024). Mass Mortality in Terns and Gulls Associated with Highly Pathogenic Avian Influenza Viruses in Caspian Sea, Kazakhstan. Viruses.

[27] Sputnik Kazakhstan Avian Flu in Kazakhstan: About 2 Million Birds Died in 2020. https://ru.sputnik.kz/20210106/ptichiy-gripp-v-kazakhstan-okolo-2-millionov-ptits-pogiblo-v-2020-godu-15935875.html.

[28] Amirgazin A., Shevtsov A., Karibayev T., Berdikulov M., Kozhakhmetova T., Syzdykova L., Ramankulov Y., Shustov A.V. (2022). Highly Pathogenic Avian Influenza Virus of the A/H5N8 Subtype, Clade 2.3.4.4b, Caused Outbreaks in Kazakhstan in 2020. PeerJ.

[29] Abenova A., Mukhanbetkaliyev Y.Y., Kadyrov A., Sytnik I.I., Shevtsov A., Korennoy F.I., Martin I.I., Perez A.M., Abdrakhmanov S.K. (2025). Environmental Suitability of Kazakhstan to Highly Pathogenic Avian Influenza Using Data on Eurasian Outbreaks, 2020–2024. Viruses.

[30] Iglesias I., Ruzmatov S., Ibañez-Porras P., Kadyrov A., Bakishev T., Korennoy F., Perez A., Abdrakhmanov S. (2025). Risk assessment of highly pathogenic Avian Influenza (HPAI) in wild bird species in Kazakhstan. Ger. J. Vet. Res..

[31] Bekshin Z., Temirbekova A., Nurbekova Z., Amirkhanova N., Satenova A., Askarov A., Zakarya K., Abduraimov Y., Rsaliyev A. (2025). Epidemiology, Virology, and Control of Highly Pathogenic Avian Influenza in Kazakhstan. Pathogens.

[32] Annual Reporting Meeting of the Members of the Union of Poultry Farmers of Kazakhstan. https://ptica.kz/news/otchetnoe-godovoe-sobranie-chlenov-ojufl-sojuz-pticevodov-kazahstana?lang=en.

[33] Malczewski J. (2006). GIS-Based Multicriteria Decision Analysis: A Survey of the Literature. Int. J. Geogr. Inf. Sci..

[34] Sullivan B.L., Wood C.L., Iliff M.J., Bonney R.E., Fink D., Kelling S. (2009). EBird: A citizen-based bird observation network in the biological sciences. Biol. Conserv..

[35] Central Veterinary Institute, Wageningen University (2017). Risk factors of primary introduction of highly pathogenic and low pathogenic avian influenza virus into European poultry holdings, considering at least material contaminated by wild birds and contact with wild birds. EFSA Support. Publ..

[36] Kazakhstan Waterways (OpenStreetMap Export). https://data.humdata.org/dataset/hotosm_kaz_waterways.

[37] JRC Global Surface Water Mapping Layers, v1.4. https://developers.google.com/earth-engine/datasets/catalog/JRC_GSW1_4_GlobalSurfaceWater.

[38] Keeler S.P., Dalton M.S., Cressler A.M., Berghaus R.D., Stallknecht D.E. (2014). Abiotic factors affecting the persistence of avian influenza virus in surface waters of waterfowl habitats. Appl. Environ. Microbiol..

[39] ERA5-Land Daily Aggregated—ECMWF Climate Reanalysis. https://developers.google.com/earth-engine/datasets/catalog/ECMWF_ERA5_LAND_DAILY_AGGR.

[40] Ibáñez-Porras P., de la Torre A., Gómez-Pérez J.I., Tomás-Tenllado C., García E., Cáceres G., Pérez A., Iglesias I. (2024). DiFLUsion: A Novel Space-Time Alert System for HPAI. Ger. J. Vet. Res..

[41] Lambert S., Durand B., Andraud M., Delacourt R., Scoizec A., Le Bouquin S., Rautureau S., Bauzile B., Guinat C., Fourtune L. (2022). Two Major Epidemics of Highly Pathogenic Avian Influenza Virus H5N8 and H5N1 in Domestic Poultry in France, 2020–2022. Transbound. Emerg. Dis..

[42] Alexakis L., Buczkowski H., Ducatez M., Fusaro A., Gonzales J.L., Kuiken T., Ståhl K., EFSA (European Food Safety Authority), ECDC (European Centre for Disease Prevention and Control) (2025). Avian Influenza Overview September–December 2024. EFSA J..

[43] Bureau of National Statistics of the Agency for Strategic Planning and Reforms of the Republic of Kazakhstan Animal Husbandry of the Republic of Kazakhstan: 2025; Bureau of National Statistics: Astana, Kazakhstan, 2025. https://stat.gov.kz/.

[44] Hwang C.L., Yoon K. (1981). Multiple Attribute Decision Making: Methods and Applications. Lecture Notes in Economics and Mathematical Systems.

[45] El Allaki F., Christensen J., Vallières A. (2019). A Modified TOPSIS (Technique for Order of Preference by Similarity to Ideal Solution) Applied to Choosing Appropriate Selection Methods in Ongoing Surveillance for Avian Influenza in Canada. Prev. Vet. Med..

[46] Saaty T.L. (1980). The Analytic Hierarchy Process: Planning, Priority Setting, Resource Allocation.

[47] Robin X., Turck N., Hainard A., Tiberti N., Lisacek F., Sanchez J.-C., Müller M. (2011). PROC: An Open-Source Package for R and S+ to Analyze and Compare ROC Curves. BMC Bioinform..

[48] Youden W.J. (1950). Index for rating diagnostic tests. Cancer.

[49] Jenks G.F. (1967). The Data Model Concept in Statistical Mapping. Int. Yearb. Cartogr..

[50] (2025). What Is the Jenks Optimization Method? (ArcGIS Knowledge Base). https://support.esri.com/en-us/knowledge-base/what-is-the-jenks-optimization-method-1462479759617-000006743.

